# Wind dynamics and leaf motion: Approaching the design of high-tech devices for energy harvesting for operation on plant leaves

**DOI:** 10.3389/fpls.2022.994429

**Published:** 2022-10-26

**Authors:** Fabian Meder, Giovanna Adele Naselli, Barbara Mazzolai

**Affiliations:** Bioinspired Soft Robotics, Istituto Italiano di Tecnologia, Genova, Italy

**Keywords:** wind damage, plants and artificial devices, biohybrid, bioinspiration, dynamic models of petiole

## Abstract

High-tech sensors, energy harvesters, and robots are increasingly being developed for operation on plant leaves. This introduces an extra load which the leaf must withstand, often under further dynamic forces like wind. Here, we took the example of mechanical energy harvesters that consist of flat artificial “leaves” fixed on the petioles of *N. oleander*, converting wind energy into electricity. We developed a combined experimental and computational approach to describe the static and dynamic mechanics of the natural and artificial leaves individually and join them together in the typical energy harvesting configuration. The model, in which the leaves are torsional springs with flexible petioles and rigid lamina deforming under the effect of gravity and wind, enables us to design the artificial device in terms of weight, flexibility, and dimensions based on the mechanical properties of the plant leaf. Moreover, it predicts the dynamic motions of the leaf–artificial leaf combination, causing the mechanical-to-electrical energy conversion at a given wind speed. The computational results were validated in dynamic experiments measuring the electrical output of the plant-hybrid energy harvester. Our approach enables us to design the artificial structure for damage-safe operation on leaves (avoiding overloading caused by the interaction between leaves and/or by the wind) and suggests how to improve the combined leaf oscillations affecting the energy harvesting performance. We furthermore discuss how the mathematical model could be extended in future works. In summary, this is a first approach to improve the adaptation of artificial devices to plants, advance their performance, and to counteract damage by mathematical modelling in the device design phase.

## Introduction

Artificial devices, sensors, energy harvesters, and even small robots have been developed for installation and operation on plants, especially on leaves (Giraldo et al., 2019; Lew et al., 2020; Dufil et al., 2022). Such devices are either permanently or transiently fixed on a plant leaf where they perform a specific task and have a potential impact on monitoring and preserving plants and ecosystems. Examples are sensors that measure plant parameters (Khan et al., 2018; Jiang et al., 2020; Diacci et al., 2021; Fiorello et al., 2021; Dufil et al., 2022); molecule delivery platforms (Fiorello et al., 2021); robots and drones that could use a plant or a leaf as support (Graule et al., 2016; Fiorello et al., 2021; Jiaming et al., 2021), and, moreover, energy harvesting artificial leaves (Jie et al., 2018; Meder et al., 2018; Meder et al., 2020a; Wu et al., 2020; Meder et al., 2021; Meder et al., 2022).

The latter have recently been shown to be capable of converting wind into electrical energy: the artificial leaves installed on plant leaves exploit the wind-induced leaf oscillations and fluttering for a mechanical-to-electrical energy conversion (Meder et al., 2020a; Meder et al., 2021). In more detail, the motion of the artificial leaf fixed firmly on the petiole of the natural leaf causes the two surfaces to contact and separate due to air flow. This produces static charges on the leaf surface (charges mainly positively) and on the artificial leaf (charges mainly negatively) through contact or tribo-electrification. When the two surfaces separate after their contact, these charges do not compensate for each other anymore and they are then electrostatically induced into the inner plant tissue (acting as an ion-conductive electrode) and into an electrode in the artificial leaf, where they can be harvested as electrical energy. This could directly power light-emitting diodes and a temperature sensor (Meder et al., 2020a; Meder et al., 2021). Parameters like impact force, impact frequency, contact area, and separation distance affect the electrical output amplitude. Thus, the motion and mechanical behavior of the system play an essential role. The artificial leaves have been engineered to be transparent and soft (elastic) to reduce harm to the tissue and not hamper photosynthesis.

The biomechanics of plant tissue under wind dynamics such as leaf fluttering has been investigated using different mechanical models (Gonzalez-Rodriguez et al., 2016; De Langre, 2019; Geitmann et al., 2019; Jackson et al., 2021; Langer et al., 2022; Lauderbaugh and Holder, 2022; Roth-Nebelsick et al., 2022). Some of them consider the effect of foreign bodies like insects on leaf mechanics (Whitney and Federle, 2013). Similarly, all artificial devices, from sensors to energy harvesters or robots specifically developed to operate on leaves, introduce a specific load on the plant, and this could be tailored during the design of the technology. A model that allows one to evaluate the dynamic behavior of leaves together with artificial devices permanently or transiently installed on them would be helpful for such a design but has not been proposed yet. As it is likely that the number and also the functionality of devices for operation on plants will increase in the future (Dufil et al., 2022), it is essential to develop an approach to better predict the mechanical effect on the tissue under the weight of the device and under the load of wind.

Here, we report the first computational approach to describe, investigate, and predict the behavior of a plant leaf with a device installed as a load on the petiole. In particular, we investigate, as an example, a leaf-coupled mechanical wind-energy harvester reported earlier (Meder et al., 2020a; Meder et al., 2021). First, the natural and the petiole of the artificial leaf are individually modeled as flexible elements that sustain their lamina, statically and dynamically deflecting under gravity and wind, respectively. Moreover, the effect of the combination of artificial leaves installed on the top of the plant leaf is modeled as a function of its dimensions, material properties, and certain wind speeds. The plant petiole carrying both its own lamina and the artificial leaf, is expectedly most prone to failure, and we evaluated the maximum additional load that it can support before damage and dropping of the leaf occurs. Furthermore, our model allows us to adapt the plant–artificial leaf biohybrid system in terms of the dimensions and materials of the artificial leaf by observing the combined oscillatory behavior and finding an optimal impact-release motion essential for energy harvesting. To retrieve the structural properties of the two leaves for the model, we suggest a set of straightforward static experiments to assess leaf deflection and oscillation under gravity and with additional weights. We used here the model species *Nerium oleander*, an ever-green plant with leathery, glabrous, elliptic, lanceolate-shaped leaves, which has been reported earlier as an excellent species for combination with artificial systems for wind-energy harvesting (Meder et al., 2020a; Meder et al., 2021). Moreover, we report the electricity produced by such a system in the wind as a function of the collective motion of the leaf and its artificial parts. The results show that the model is a step towards a platform to design and especially dimension artificial devices for operation on plants.

## Materials and methods

### Overview of the proposed approach

**Figure 1:**
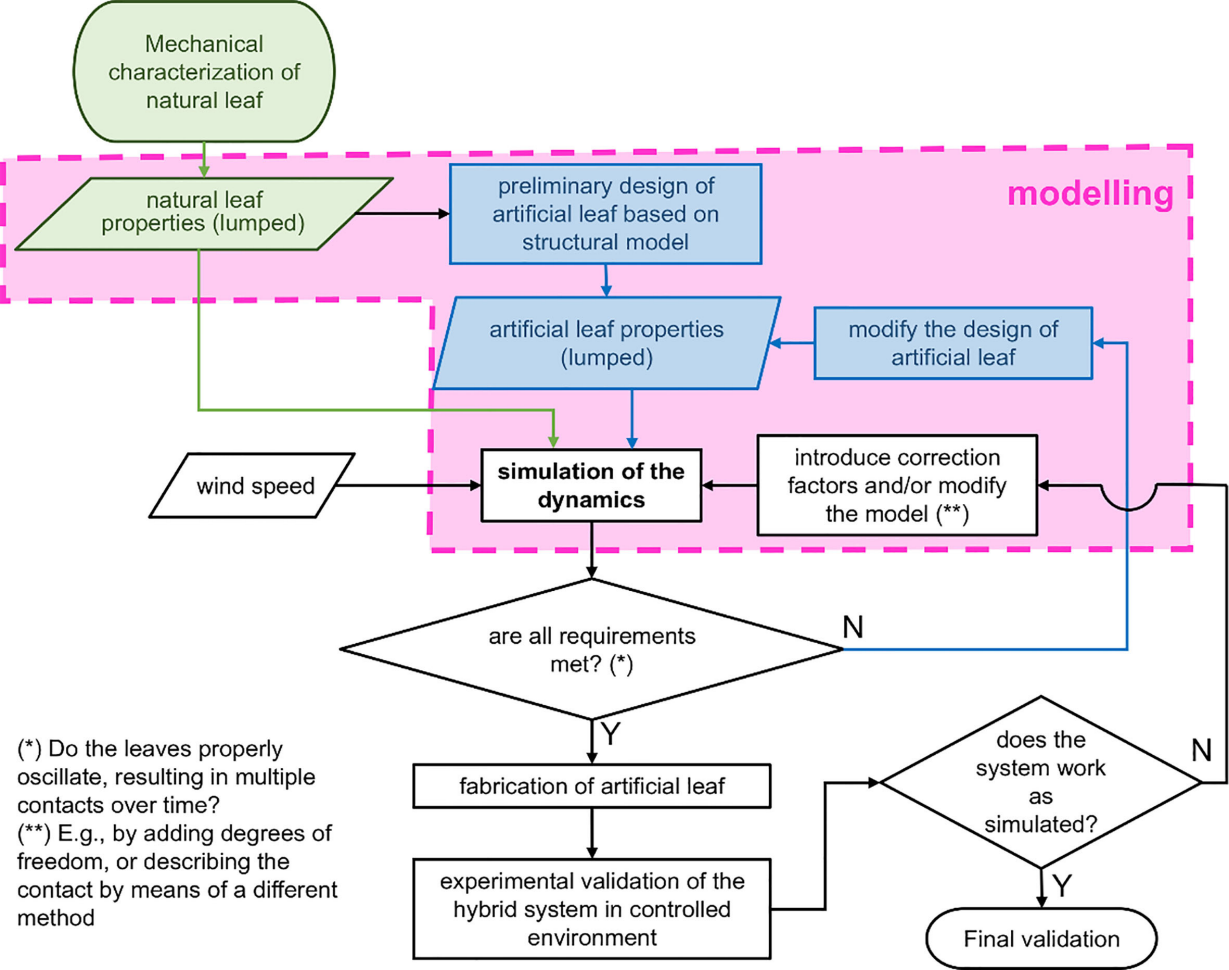
Flowchart of the proposed approach to design and develop plant-hybrid energy harvesters. Green and blue blocks refer to natural and artificial leaves, respectively. The pink shape contains the modeling stages.

**Figure 1** shows a flowchart that summarizes the workflow of the proposed approach. All steps related to modeling are enclosed by the pink rectangle. Depending on plant species, the stiffness, shape, size, and resistance of the leaves and petioles to external static and dynamic loads may differ largely. Hence, it is crucial to begin with experimentally assessing a set of mechanical parameters required for modeling of the plant leaf as described in detail later (this can also be done for the artificial leaf if previous prototypes exist, as in our case, or with a preliminary prototype obtained from the structural model). This results, if possible, in a few “lumped” parameters to approximate their spatially distributed properties at one concentrated point. These parameters serve as inputs to model the dynamics of either the single leaf or the biohybrid system under assigned external conditions. The results of the model enabled them to estimate how the hybrid system moves and oscillates and how the artificial part could be redesigned to achieve a desired behavior. To validate and adapt the model, experimental verification either in a laboratory or outdoors, can serve to foster the success of the design process. The model helps to reduce the number of prototypes needed to develop an efficiently working system. This may in turn be particularly useful in the case of devices having a relatively complex structure and when expensive or time-consuming, experimental analysis should be performed only on the most promising prototypes.

### Plants

*N. oleander* plants have been purchased at a local nursery (plant size about 1 m in height) and the plants were kept in pots outdoors. For performing the mechanical test, about 5 cm long sections of the stem containing a single leaf were cut directly before performing the experiments described below.

### Artificial leaf

The artificial leaf is a multilayered structure with a shape that resembles the leaf of the plant. Our preliminary prototype has a thin, 5 mm and about 15 mm long, base (resembling the petiole), which then expands with a fillet to a width of 30 mm and a length of 105 mm, tapered in the last 10 mm with a triangular tip (resembling the shape of the leaf lamina). It is installed on top of the plant leaf, fixing the petiole of the artificial leaf to the plant leaf petiole at approximately the first 5 mm of the petiole, here using silicone rubber tape. It serves to enhance charge generation during wind-driven contact and separation between the plant leaf and the silicone layer for triboelectric energy conversion. It is composed of three layers: a 200 µm polyethylene terephthalate (PET) layer at the top, a 50–100 nm indium tin oxide (ITO) layer in the middle, and a 500 µm silicone rubber layer (Shore A 60) at the bottom. The total weight is 3 g. Its structure and properties regarding energy conversion together with plants have been described in detail in Meder et al. (2020a).

### Experimental assessment of leaf mechanical properties in static and dynamic tests

First, a straightforward mechanical characterization to derive the lumped parameters for both the artificial and natural leaves was performed in a dedicated setup in which a ~5 cm section of the stem with a single leaf was mechanically fixed about 2 cm above and below the petiole onto a stiff column so that the leaf could freely oscillate. During the static tests, the leaf was dropped from a horizontal position and oscillations were tracked until equilibrium was reached. For tracking, video recordings (such as shown in **Supporting Video 1**) were analyzed with the software Tracker (version 5.0.7) tracking markers on the tip, center, and end (close to the beginning of the petiole) of the leaf blade. The same tests have been performed on the artificial leaves. Quasistatic tests with seven individual leaves from the same plant were compared here.

Second, the maximum load the natural petiole can sustain was assessed by applying a gradually increasing load at the center of the leaf blade until the petiole failed while tracking the deformation of the leaf lamina under the load (**Supplementary Figure S1** shows the details of the straightforward experimental setup).

Third, dynamic tests have been performed, exposing the leaves to air flow. To simulate wind, an air flow was created by a brushless cooling fan with an outlet diameter of 4 cm or a compressed air source with an outlet diameter of 0.7 cm at a distance of about 100 cm from the leaf under investigation. The flow was controlled by adjusting the supply voltage of the fan, and resulting wind speeds were measured using a hot wire anemometer (405i, Testo SE & Co. KGaA, Germany) at a distance of ~2 cm in front of the leaf. The adjustable average wind speeds range from ~1 to 8 m/s. The angle between the surface of the leaf and the axis of the outlet of the fan is approximately 90°. We did not rigorously control this angle. Although it is a crucial quantity in aerodynamics, our approach is motivated by the fact that biohybrid energy harvesters should be designed to operate outdoors, where conditions such as the direction of the wind will vary and are often unknown *a priori*. Dynamic tests in air flow have been performed using four replicates (four leaves of two different plants) at three different wind speeds each.

### Characterization of energy conversion during dynamic tests

Current, voltage, and charge measurements have been performed using a high input impedance electrometer (6517B, Keithley, USA) and an oscilloscope (MSO7014A, Agilent Technologies, USA). High-speed video recordings have been done using a camera (MC1302, EOSense, Mikrotron GmbH, Germany) at typically 900 frames per second (fps).

### Mechanical model: Statics and dynamics

**Figure 2:**
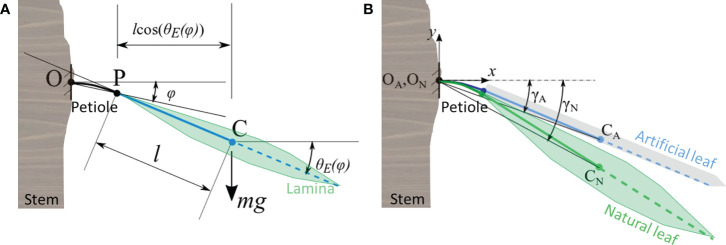
Sketch of the mathematical model and its parameters and variables: **(A)** representation of the generic leaf fixed at point O at the stem having a single degree of freedom (angle φ). We assume that the transition from petiole to lamina occurs at point P and that the mass m of the lamina with half-length l is concentrated at its center of mass C; *θ_E_* is the rotation of the free end related to the angle *ϕ*, g is the gravitational acceleration. The natural leaf is depicted, but the description is valid for the artificial leaf as well. **(B)** Schematic of a plant leaf (subscript N) with an artificial leaf (subscript A) installed on top of it, both fixed at the branch with their petioles at points O_N_ and O_A_, respectively. The angles *γ_A_* and *γ_N_* are computed to ensure that interpenetration of the two leaves cannot occur.

This work represents the first steps towards developing design methods for hybrid energy harvesting systems operating on plant leaves. Thus, we start by proposing a model with a low number of degrees of freedom. Keeping this in mind, we model a leaf as a torsional spring, under the assumption that it performs only planar bending in the vertical plane. The approach is the same for both natural and artificial leaves. We treat the petiole as a flexible body and approximate the lamina as a rigid body, that is, we assume that the compliance of the leaf is localized at the petiole. A similar approach is found in Lauderbaugh et al. (2021), with the difference that we account here for large deflections. To do this, we applied a technique named nonlinear matrix structural analysis (NMSA), as in our previous work (Naselli and Mazzolai, 2019). In short, the technique consists of discretizing the petiole as a series of segments, each being a flexible element with two end points (called *nodes*), and computing its deformation when acted upon by a set of external loads (called *nodal forces*). The stiffness of the petiole is described by a stiffness matrix. For a more comprehensive and detailed explanation of NMSA, the reader can refer to (McGuire et al., 2000). In this work, the configuration of the leaf is described by a single degree of freedom: the angle *φ* as shown in **Figure 2A**. It is measured positive counterclockwise from the horizontal line, between the horizontal plane and the line passing through the two end points of the petiole. The rotation angle of the section at the apex of the petiole (point P), *θ_E_*, is a function of *φ*. At their basal points, *O_A_* and *O_N_*, the petioles are fully constrained and considered fixed in space.

The lamina is simulated as a combination of a vertical force and a moment due to the distance *l* between the apex P and the center of mass of the lamina, C. Loads are applied incrementally to account for the large deflection undergone by the petiole. Then, the rotation *θ_E_* of the last element of the discretization (that is, the free end) is related to the angle *φ*. At the same time, we relate the applied moment to the same angle to derive the value of the lumped torsional stiffness of the petiole, *K_t_*. Although not rigorous, this approach allows one to fictitiously account for the effect of the gravity force while using a torsional spring in the model. Moreover, this approach allows to model a nonlinear relation between *φ* and the applied moment, if needed.

The equation of motion of a leaf oscillating under the effect of gravity until it reaches its static deflection at equilibrium, is:


(1) $$J_{t}\ddot{\varphi }+R\dot{\varphi }+K_{t}\varphi =mgl\cos\left(\theta_{E}\left(\varphi \right)\right)$$

In which *J_t_* = *ml* ^2^ is the moment of inertia, and *R* denotes the structural damping, that can be estimated either on the basis of the experimental test described in *Experimental assessment of leaf mechanical properties in static and dynamic tests*, or on previous knowledge of the material properties of the leaf. Accounting for the effect of the wind on the oscillation of the leaf is accomplished by summing on the right side of the equation the term:


(2) $$M_{wind}=\frac{1}{2}\rho_{air}SlV_{r}^{2}\cdot \left(-C_{D}\sin\psi +C_{L}\cos\psi \right)$$

Where *ρ*_air_ = 1.22 kg/m^3^ is the air density, *S* the surface of the lamina, *V_r_* is the relative velocity of the flow, ψ the angle of attack, and *C_D_* and *C_L_* the drag and the lift coefficients (that we obtained from Ortiz et al. (2015), respectively, and they are both functions of ψ. The angle ψ, notably, depends on the first derivative of *φ* (see **Equation S5**). The equations above are valid for both leaves. From now on, the subscripts A and N will be used to refer to the artificial and natural leaf, respectively.

For both the artificial and natural leaves, the same modeling stages took place: (*i*) we modeled the petiole by NMSA, from which we obtained the lumped stiffness *K_t_* and an expression for *θ_E_* (*φ*); (*ii*) we validated the lumped stiffness and inertia of the leaf by using Equation 1, based on comparison with the experimental results (see corresponding results in Sections *Quasi-static tests on N. oleander leaves and estimation of potential damage in the hybrid system* and *Leaves lumped parameters for the equations of motion*). Then (*iii*), we modeled the dynamics of the hybrid system under the action of the wind, to investigate how the mechanical properties and the characteristics of the wind influence the oscillating behavior. This is especially interesting for the energy harvesting mechanism as it gives information on whether the artificial and natural leaves contact each other under certain conditions, hence enabling contact electrification of the two surfaces which produce the charges during energy conversion. Therefore, it is necessary to introduce additional terms in the equations of motion to account for the occurrence of the impacts. There are multiple methods to model the contact between two bodies. Here, for simplicity, we use a penalty method as described in Tornambè (1999), imposing only one condition: the artificial leaf must always remain on top of the natural one. This is done by introducing a fictitious spring between the leaves, with null stiffness when the leaves are not in contact, and positive stiffness when the two leaves tend to penetrate each other. Given the two angles *γ_A_* and *γ_N_* (as in **Figure 2**, in which they are negative), it must be


(3) $$\gamma_{A}\left(\varphi_{A}\right)-\gamma_{N}\left(\varphi_{N}\right)\geq 0$$

being


(4) $$\begin{array}{c} \gamma_{J}\left(\varphi_{J}\right)=\tan^{-1}\frac{y_{C_{J}}}{x_{C_{J}}}\\ x_{C_{J}}=l_{P_{J}}\cos\varphi_{J}+l_{J}\cos\theta_{E_{J}}\left(\varphi_{J}\right)\\ y_{C_{J}}=l_{P_{J}}\sin\varphi_{J}+l_{J}\sin\theta_{E_{J}}\left(\varphi_{J}\right) \end{array}$$

with *J=A,N*, and *l_p_* denoting the petiole length. Details of the implementation are given in the **Supplementary Information**.

Concerning the modeling stages (*i*) and (*ii*), it is worth noting that our approach is conceptually different from that reported in Lauderbaugh et al. (2021): instead of obtaining the mechanical properties from the experiment, we decided to estimate them with a mechanical model based on the material and geometrical properties of the leaf. The reason is that, in this manner, we can use our model as a tool for design purposes, for example, to estimate whether it is convenient to increase (or to decrease) the thickness of the artificial petiole to enhance the performance of the biohybrid system.

In the following, the specific characteristics of the two leaves in relation to their model are described in detail. Moreover, we address their mechanical resistance, as it must be ensured that the coupling between the artificial leaf and its natural counterpart does not lead to damage.

The entire model is implemented in the MATLAB^®^ environment and the equations of motion are solved numerically by means of the built-in solver *ode23s*.

#### Artificial leaf: Modeling, properties, and damage

Prior to modeling the dynamics, the static behavior of the artificial leaf needs to be analyzed. As said, the artificial leaf is a multilayer composed of three layers (200 µm PET, ~50–100 nm ITO, and 500 µm silicone rubber, Shore A 60). We assume that the PET layer, which most determines the stiffness of the artificial leaf, is its stiffest component and therefore it is the only layer modeled as a spring. The nm-thin ITO electrode does not significantly affect either the mass or the stiffness; the silicone rubber layer, instead, provides a negligible stiffness but a significant mass (see **Supplementary Information Section 2** for more details).

To perform NMSA, the 10 mm long artificial petiole is discretized in 6 elements of equal length, having thickness *t*_*P*_*A*__=  0.2 mm, and width *b*_*P*_*A*__=  4 mm, except the two apical elements to which we impose 6 mm and 8 mm width, respectively to account for the fillet. We fully constrain the base, and we apply the combination of two loads at the free end as already described above. The vertical displacement of point C_A_ is calculated as


(5) $$y_{C_{A}}\left(t\right)=l_{P_{A}}\sin\varphi \left(t\right)+l_{A}\sin\theta_{E_{A}}\left(\varphi \left(t\right)\right)$$

As this is our first attempt at the dynamic modeling of the leaf, the damping coefficient *R_A_* has been estimated based on the experimental results by means of the logarithmic decrement.

#### *N. oleander* leaf

**Figure 3:**
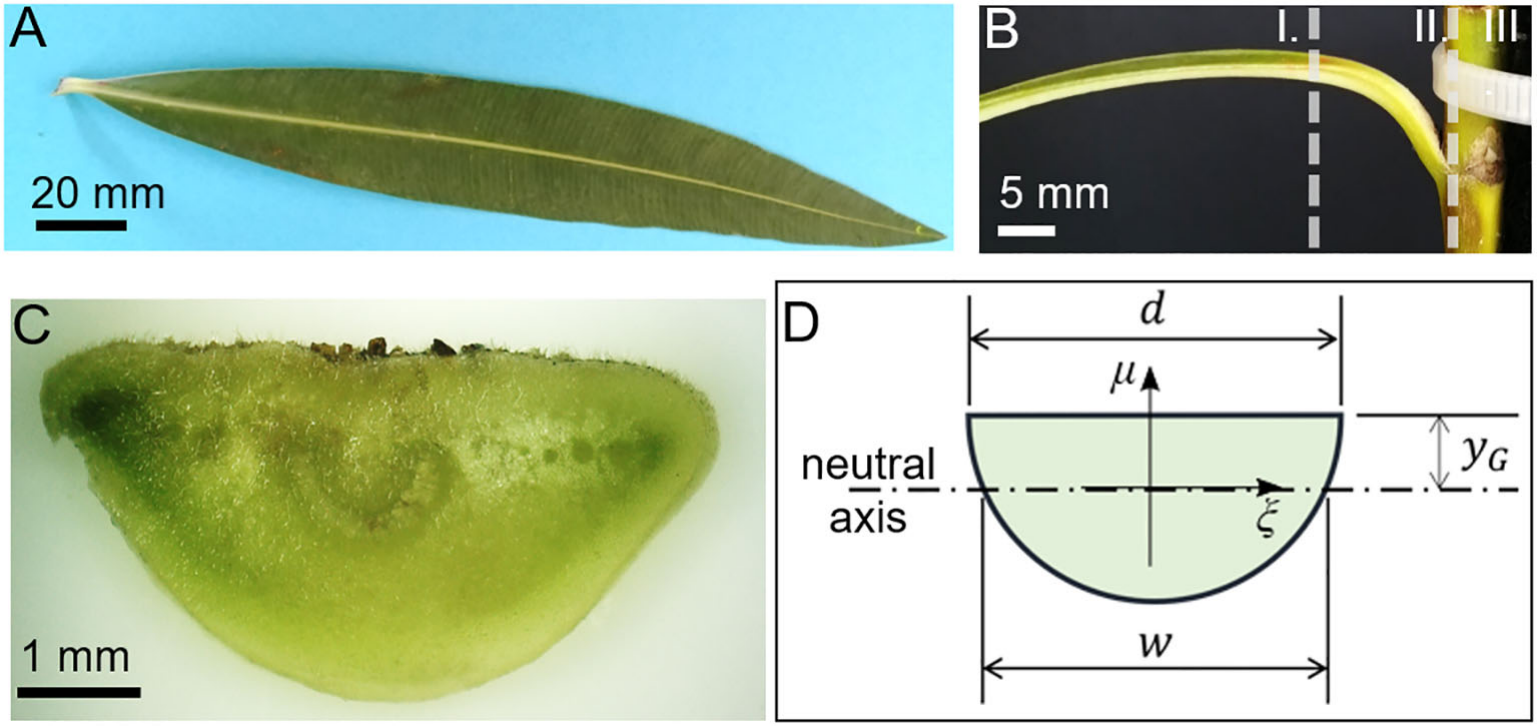
Leaf of *Nerium oleander*. **(A)** Leaf with a short petiole and a lanceolate lamina with a prominent middle vein. **(B)** From the stem arises a petiole that is curved like a quarter circle with lamina **(I)**, petiole **(II)**, and stem **(III)**. **(C)** Digital microscopy of an unstained transverse section of the petiole with a flat adaxial (= upper) side and a convex abaxial (=lower) side close to the base where it is attached to the stem. **(D)** Model of the petiole assuming a semicircular cross-section and relevant parameters.

*N. oleander* is used as a model species due to its previously shown suitability for biohybrid wind-energy harvesting as mentioned above (Meder et al., 2020a; Meder et al., 2020b). **Figures 3A, B** show the top and side view of the leaf, respectively. For simplicity, we model the cross-section of the petiole in **Figure 3C** as the semicircle in **Figure 3D**, with the diameter *d* resembling the *N. oleander* properties. This geometry may need to be varied for other plant species as petiole geometries may differ (Pasini and Mirjalili, 2006).

In our case, the distance *γ_G_* from the center of the circle and the center of mass is 2*d*/3*π*; the width *w* at the neutral axis is $2\sqrt{\frac{d^{2}}{4}-y_{G}^{2}}$ . By treating a weight attached to the petiole as a load *V* that acts vertically on it, according to the Jourawski formula, the maximum shear stress at this section can be computed as *τ* = *VQ*/*wI*, in which *I* denotes the second moment of inertia of the section and *Q* is the static moment of the section above the neutral axis. For the case of a semicircle, from Steiner’s theorem we obtain $I=\pi d^{4}/128-Ay_{G}^{2}$ , being A the area, and the static moment Q is calculated as


(6) $$Q=\int_{-d/2}^{d/2}d\xi \int_{0}^{y_{G}}d\mu \mu -\;\;2\int_{\frac{w}{2}}^{\frac{d}{2}}d\xi \int_{0}^{y_{G}-\sqrt{\frac{d^{2}}{4}-x^{2}}}d\mu \mu$$

A value *V_U_* that describes a load that will cause the fracture of the petiole is thus related to the maximum shear stress,*τ*_*U*_ , that the leaf can support.

To compute the stiffness of the natural leaf, we adopted NMSA as done for the artificial one, with the main difference that the longitudinal axis of the petiole is described as a circular arc spanning 90°. The torsional stiffness *K*_*t*_*N*__ is then derived from NMSA, assuming that the natural petiole has a Young’s modulus equal to 20 MPa [a value based on Ciupak et al. (2019)]. It is worth remarking that here we have neglected the fact that petioles are not homogeneous and isotropic structures. This is the reason why their bending and tensile modulus derived by biomechanical tests can differ, sometimes largely [See, e.g., Langer et al. (2021a; 2021b)]. An advantage of our approach, based on NMSA, is that it allows us to model possible non-homogeneities that cannot be ignored. For example, a cross-section composed of tissues with different properties, can be modeled as a system composed of two springs in parallel, each having a stiffness matrix.

## Results

### Structural properties of the biohybrid system for wind-energy harvesting

**Figure 4:**
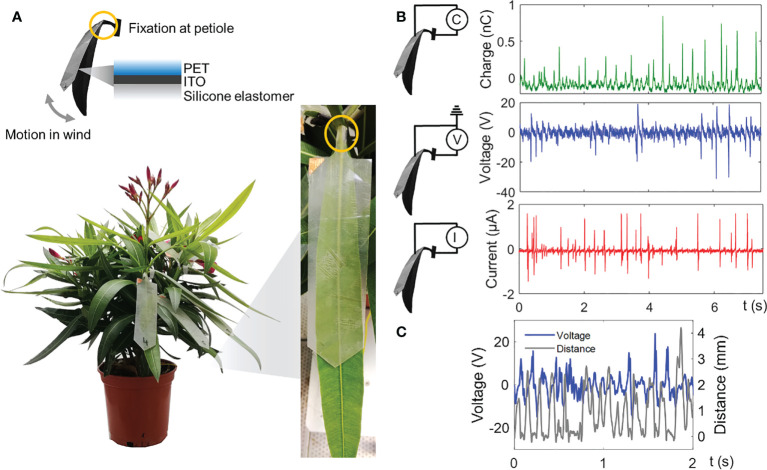
Components and electrical measurements with a plant-hybrid wind-energy harvester. **(A)** Typical configuration of a *N. oleander* plant modified with artificial leaves for wind-energy harvesting exploiting motion of leaves in the wind. **(B)** Charge, voltage, and current signals were generated by a single natural leaf–artificial leaf combination at an average wind speed of 4.1 ± 0.3 m/s. The signal spikes resemble the contact and separation motion of the leaf. The measurements shown are representative measurements for four leaves tested. **(C)** Simultaneous measurement of the voltage and the distance between the artificial leaf and the plant leaf shows that voltage signals correspond to contact (distance = 0 mm) and separation motion of the two leaves.

**Figure 4A** shows a typical configuration of a plant-hybrid installation used for wind-energy harvesting. An artificial leaf, as further described in the *Materials and methods* section, is installed on the plant leaf and firmly fixed on its petiole so that the laminae can freely move under wind excitation. **Supporting Video 2** shows a high-speed camera recording of the plant-hybrid system in air flow. **Figure 4B** demonstrates typical charge (up to 0.9 nC), voltage (reaching over 20 V), and current (1–2 µA spikes) measurements obtained from the leaf interactions in air flow at a speed of ~4 m/s. The current and voltage spikes, generated by the repeating contact and separation between the leaf and the artificial leaf, follow the pattern of the leaf motion and oscillations as they are directly correlated. **Figure 4C** shows the similarity between the voltage and the tracked separation distance of the two leaves: positive voltage spikes start to form almost instantaneously when the surfaces separate after contact (0 mm distance). Further detailed analysis of this correlation is given in Meder et al. (2020a; 2021). The results show that contact and separation movements due to a random wind excitation occur irregularly, here with a frequency of about 5–7 Hz during operation at 4 m/s air flow.

### Quasi-static tests on *N. oleander* leaves and estimation of potential damage in the hybrid system

Next, it is essential to estimate the maximum load the artificial leaf could apply to the natural petiole to predict if it could damage it. An initial experimental mechanical analysis of the leaves contains straightforward tests from which essential input parameters for the model can be obtained. These can be easily adapted to other plant species when performed as described in *Experimental assessment of leaf mechanical properties in static and dynamic tests*. **Table 1** reports the petiole diameter *d* of *N. oleander* leaves (n = 7), the full leaf mass *m_l_*, *m_add_* which is the mass that the leaf held before breaking as detailed below, and*τ*_*U*_ the shear stress (computed as described in *Mechanical model: Statics and dynamics*) under the shear load *V*=*m_add_g*, being *g* the gravitational acceleration (9.806 m/s²). We tested a set of seven leaves from the same plant and to evaluate the leaf damage in terms of a too high load applied to it, *m_add_* was determined by exposing the leaves to different weights. The results show that a single *N. oleander* leaf can sustain a great multiple (up to 400 times) of its own weight without detaching from the branch and/or suffering any significant damage.

**Table 1:** Petiole diameter *d*, the full leaf mass *m_l_*, mass sustained before breaking *m_add_*, and the shear stress*τ*_*U*_ determined in tests of seven *N. oleander* leaves (n = 7).

	*d* (mm)	*m_l_* (g)	*m_add_* (g)	*τ*_*U*_ (MPa)
Leaf 1	3.4	1.53	222.5	0.67
Leaf 2	3.4	1.7	188.5	0.57
Leaf 3	3.6	1.55	246.5	0.66
Leaf 4	3.7	1.74	327	0.84
Leaf 5	3.5	1.04	426	1.22
Leaf 6	3.0	0.73	180	0.7
Leaf 7	3.1	0.71	146	0.53
Mean	3.4	1.29	248.1	0.74
Standard deviation	0.3	0.45	97.6	0.23
IQR, min value	3.0	0.71	146.0	0.53
IQR, 25th percentile	3.3	0.89	184.3	0.62
Median	3.4	1.53	222.5	0.67
IQR, 75th percentile	3.6	1.63	286.8	0.77
IQR, max value	3.7	1.74	426.0	1.22

IQR, interquartile range.

From these investigations, the average ultimate shear stress in the case of *N. oleander* is 0.74 MPa, which is within a similar order of magnitude to the values reported in the literature for other plant species (see, e.g., Onoda et al. (2011); Song et al. (2022) and Ciupak et al. (2019)). For all the leaves, the fracture occurred at the connection of the petiole to the branch, as expected, as in this region local stress peaks can occur due to the transition from the branch to the petiole. Based on these results, it is safe to assume that the artificial leaf (due to its additional weight) cannot cause the fracture of the natural petiole as its weight of ~3 g is about 50 to 150 times less than *m_add_*. Although this holds for the biohybrid system based on *N. oleander*, it is certainly not the same for all plants, especially those with thinner petioles that are expected to withstand smaller maximum loads. Using other plant species requires performing the static test reported here to assess the mechanical resistance of the natural leaf before designing the artificial leaf.

We furthermore performed a stress-strain analysis of the petiole, which is especially interesting to evaluate the stability of the thin electrode layer in the artificial leaf (see **Supporting Information Section 4**). This allows one to predict the maximal bending curvature before this electrode cracks, leading to a loss of conductivity and reducing the capability to harvest charges on the artificial leaf. Indeed, the ~100 nm thick ITO electrode is the most sensitive compartment of the multilayered artificial leaf, having a Young’s modulus of ~100 GPa at room temperature and an ultimate strain of 0.022 (Cairns et al., 2001).

### Leaves lumped parameters for the equations of motion

**Figure 5:**
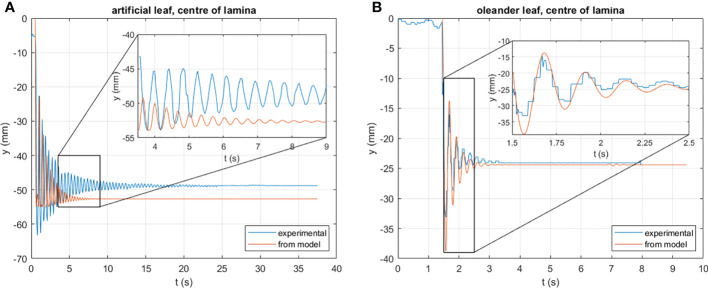
Vertical deflection *γ* of the center of the leaf under the action of gravity vs. time, as computed by the model (orange line) and by experiment; **(A)** results obtained for the artificial leaf; **(B)** results obtained for the natural leaf.

In order to obtain lumped parameters for the equations of motion of the leaves, the leaf oscillations were observed under gravity when the lamina is dropped from a horizontal position (**Supporting Video 1**). Results for the artificial leaf are shown in **Figure 5A** and for the natural leaf in **Figure 5B**, respectively.

Both graphs compare experimental data with the simulation using the parameters reported in **Table 2** to describe the artificial and natural leaf, clearly showing the similarity of the obtained oscillation period and the damping pattern, confirming that the structural modeling as described here is legitimate. Deviations are caused by nonlinear material properties and/or inhomogeneities in the structures that are not considered here. This is particularly evident with the artificial leaf. **Figure 5A**, in fact, shows that the frequency of the oscillation resulting from the model (almost 4 Hz) is slightly larger than the experimental (about 3.5 Hz). Moreover, the further discrepancy must be introduced by the flexibility of the lamina, which is not modeled here, but certainly plays a role in the displacement of its center point. This aspect, and the mentioned nonlinearities, complicate the modeling of the petiole by a few constant lumped parameters. On the one hand, further improvements to the model are advisable, based on thorough mechanical tests of the structures and on a higher number of degrees of freedom, as discussed later. On the other hand, it must be considered that, in operational conditions, the vertical deflection of the artificial leaf is limited by the presence of the natural one. Hence, we suggest that it is better to implement a model that captures the dynamic behavior in the range of interest well enough, rather than focus on extremely large deflections. The inset in **Figure 5B**, instead, shows that the adopted lumped properties allow one to model the dynamics of the natural leaf with very good agreement with the experimental results. In fact, the oscillation is well described in both amplitude and frequency (~5 Hz). However, this does not mean that, in general, natural leaves are easier to model than their artificial counterparts; this strongly depends on the specific plant species considered.

**Table 2:** Lumped parameters of artificial and natural leaves.

	*J_t_* (kg ∗ m^2^)	*R* (kg ∗ m^2^/s)	*K_t_* (Nm)	*θ_E_* (*φ*)
Artificial leaf	9.3 · 10^−6^	7.8 · 10^−6^	2.3 · 10^−4^	1.44*φ_A_*
Natural leaf	3.2 · 10^−6^	2.2 · 10^−5^	2.1 · 10^−3^	1.68*φ_N_*

J_t_, R, and K_t_ denote the inertia, damping and stiffness of the leaf, respectively required in the equations of motion. The last column contains the adopted relation between the rotation angle of the apex of the petiole and the degree of freedom used to describe the configuration of the leaf.

### Dynamics of plant leaf–artificial leaf hybrid energy harvesting system

Exploiting our model, we have investigated the dynamics of the plant-hybrid system under various conditions. First, the wind speed is a crucial parameter expected to play a major role in the overall behavior. Second, the properties of the artificial leaf can be tuned by design to affect the motion of the combined plant-hybrid system and enhance the performance of the energy harvesting system. Therefore, we performed simulations using five average wind speeds *U*, as well as varying the mass and stiffness of the petiole of the artificial leaf. The latter was done by rescaling the properties of the current artificial leaf by multiplying factors, *µ* and κ, for the mass and stiffness, respectively:


(7) $$\begin{array}{c} U\in \left\{\begin{array}{ccccc} 4, & 5, & 6, & 8, & 10 \end{array}\right\}\;m/s\\ \mu =\left\{\begin{array}{ccc} 0.6, & 1, & 1.4 \end{array}\right\}\\ \kappa =\left\{\begin{array}{ccc} 0.6, & 1, & 1.4 \end{array}\right\} \end{array}$$

Consequently, a total of 45 simulations were performed. Apart from these quantities, all the simulations share the same settings; the simulated natural leaf is kept the same, as well as the initial relative angle between the leaf and the air flow. To simulate a non-uniform air flow—as real wind is rarely uniform—the wind velocity is set as:


(8) $$\tilde{U}\left(t\right)=U\cdot (1+0.2\sin\frac{2\pi t}{T_{U}})$$

with a period *T*_*U*_ = 2 s. The initial relative angle between the leaf and the air flow is *ψ*_0_*N*__=  6° and *ψ*_0_*A*__=  12° for the natural and the artificial leaf, respectively.

**Figure 6:**
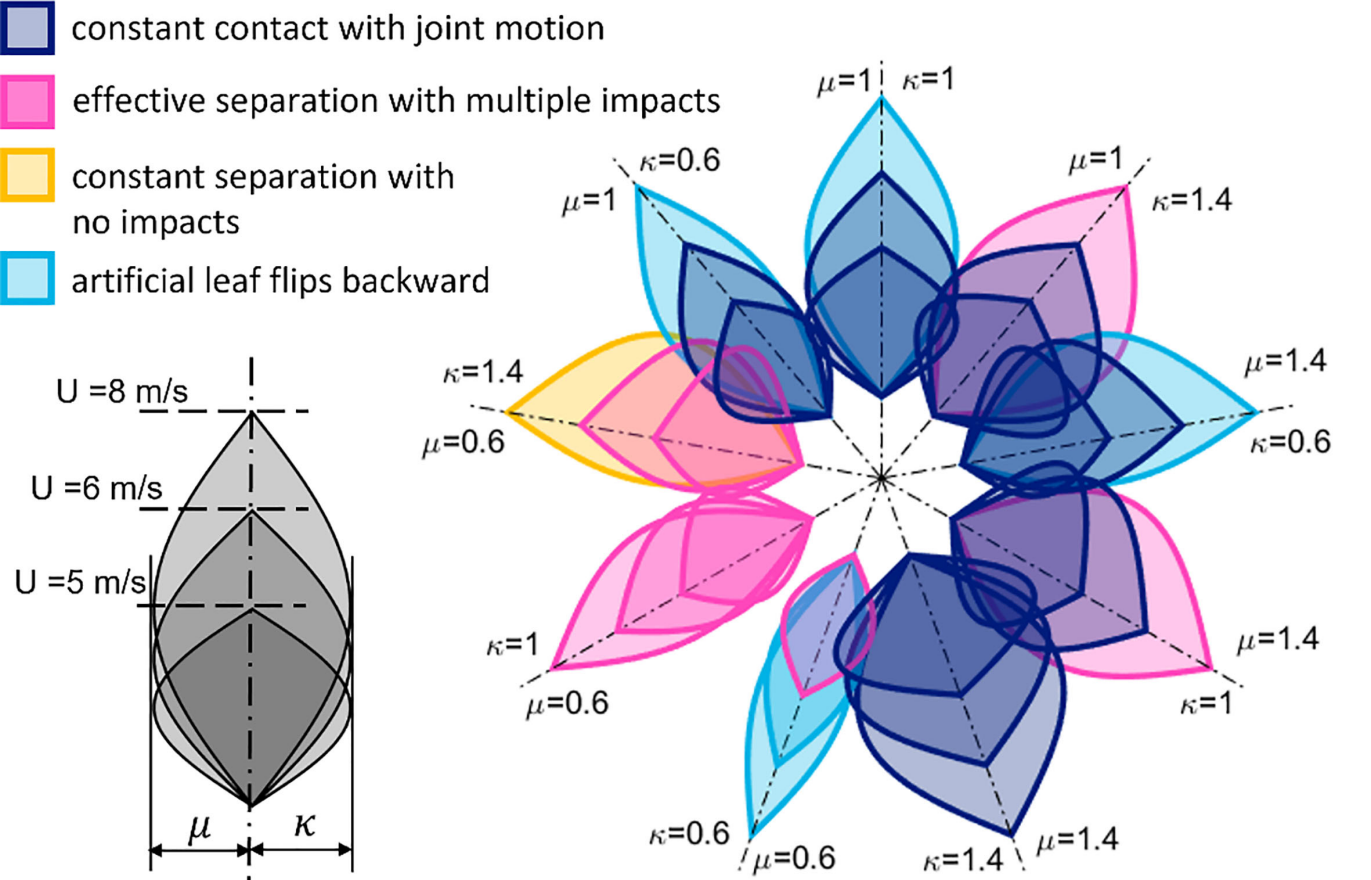
Results obtained by the dynamic model of the plant-hybrid system (natural + artificial leaf) represented as a schematic flower. Each schematic petal corresponds to a specific simulation. Different lengths of the petals correspond to different average wind speeds, as indicated in the gray sketch on the left. Petals aligned on the same axis (dash-dotted line) correspond to simulations with the artificial leaf having mass *µm_A_* and stiffness*κ K*_*t*_*A*__ in the reported values. The color of the schematic petal indicates the resulting behavior of the simulated plant-hybrid system (leaf + artificial leaf), as further specified in the legend. Results of simulations with wind speeds of 4 and 10 m/s are not shown for clarity and are described in the main text.

**Figure 6** summarizes the results of all simulations represented as a schematic flower. Each schematic petal corresponds to a specific simulation. The length of the schematic petal denotes the average wind speed. The widths of the schematic petal on the left and on the right side represent the scaling factors *µ* and κ of the mass and stiffness, respectively. The color of the petal, finally, indicates the behavior of the plant-hybrid system (leaf + artificial leaf) predicted by the simulation. The case with *µ* = 1 and κ = 1 corresponds to the artificial leaf employed so far in the here shown and previous experimental tests.

Four different behaviors occurred: (i) both leaves move jointly, constantly in contact (dark blue); (ii) the wind-induced oscillations cause effective contact and separation motion between the leaves, which results in multiple impacts (pink); (iii) the wind keeps the two leaves constantly separated, resulting in no impacts (yellow), and (iv), the artificial leaf flips backward (light blue), jeopardizing the operation of the system. For clarity, results obtained for U = 4 m/s are not displayed. At this wind speed, regardless of *µ* and κ, the two leaves were found in constant contact with joint motion. For the same reason, the results obtained for U = 10 m/s are not given in the figure as either the leaves never collided, or the artificial leaf flipped backward, thus indicating conditions that would not be beneficial for causing the ideal contact-separation motion required for good performance of the energy harvester. However, the graph clearly shows that the mass and stiffness of the artificial leaf affect the dynamic behavior of the plant-hybrid system and that they can be used to tune its motion behavior and adapt it to the typical wind speeds in the environment. Moreover, the simulations suggest that even designs could be found in which multiple impacts with effective separation occur for all displayed wind speeds (here *µ* = 0.6 and κ = 1). **Supporting Video 3** shows the simulated dynamics of the “leaf–artificial leaf combination” at 6 m/s. Although the artificial leaf previously investigated was able to produce electricity at different wind speeds, the simulations for this leaf (*µ* = 1 and κ = 1) suggest that either constant contact or flipping backwards would occur. Indeed, it can be observed from **Supporting Video 2** that the petioles remain in contact (in the experiment, it is U = 4 m/s). However, here, the flexibility of both laminae causes contact and release motion, leading to the electrical signals reported in **Figure 4**. This suggests that properties such as the flexibility of the lamina, the torsional oscillations, and the irregular air flow, not yet considered in the equations, should be included in the future to better describe the complex plant-hybrid system as further detailed in the *Discussion*. Yet, the results clearly indicate that a lighter artificial leaf could be the most promising in energy harvesting experiments as a design strategy for future prototypes to be chosen for experimental validation.

## Discussion

The results highlight key aspects to consider when dimensioning high-tech devices for operation on leaves, especially in the case of mechanical energy harvesters installed on the leaves. First of all, the investigations show that *N. oleander* is an excellent candidate to implement such systems for at least two reasons. The *N. oleander* petioles are able to withstand a great multiple of the weight of the leaf without causing damage or leaf dropping. Indeed, in principle, there are two weak points where the leaf system could be damaged: the transition between the petiole and the lamina and the transition between the petiole and the stem. The experiments showed that the weakest point for the hybrid systems seems to be the petiole–stem transition, as additional load led to a failure there. Nevertheless, in our static tests, we found that the petiole of *N. oleander* can sustain a relevant multiple of the mass of the leaf before collapsing. In other words, it can be said that the safety factor of the connection of the leaf to the branch is extremely high; it ranges from about 111 to 409 when calculated as *m_add_*/*m_l_*. Even considering the drag force exerted by the wind, it is safe to assume that the wind alone is not sufficient to detach the leaf from its branch. For example, assuming a wind speed equal to 10 m/s acting on a leaf having a surface of ~0.0026 m^2^ (that is an area computed as 0.13 × 0.02 m), and taking the density of air as 1.2 kg/m^3^, the drag force would result in only 0.156 N, corresponding to an additional load of 16 g applied on the leaf. Individual leaves of *N. oleander* seem to differ significantly from what has been observed for other plants for which safety factors are notably lower, as for example, reported in detail in Langer et al. (2021a).

Consequently, the analysis shows that neither the load of the artificial leaf in its current design (mass of 3 g) nor the forces it may exert on the leaf lead to substantial petiole damage under the tested conditions (excluding major torsional motion at this stage that has not been observed in experiments so far). This, as mentioned before, may change if other species with different biomechanical properties are used. However, the work flow given in **Figure 1** can be adopted for other species. It would be also interesting to include the petiole transition zones (at stem and lamina) with geometric and mechanical properties differing from the petiole in the model to better describe the behavior provided that the data on the structure are available, either from the literature or obtained by mechanical characterization. Recent studies showed that the damage-resistant petiole–lamina transition zone is indeed an overlooked but essential part of the foliage leaves, as for example, the twist-to-bend ratio (flexural rigidity divided by the torsional rigidity) varies along the structure (Sacher et al., 2019; Langer et al., 2021b).

The prediction of the plant-hybrid system under dynamic conditions, especially in wind, is significantly complex. The major aspect in designing biohybrid energy harvesters to be used outdoors is that they are exposed to conditions that may vary widely. The direction of the wind is not always the same, which means that the orientation between the leaves and the air flow is variable; moreover, the wind speed is hardly perfectly constant—at least, not over long periods of time. Moreover, leaf properties, especially size, shape, turgor pressure, and hence stiffness, likely vary, even on the same plant. Such unstructured operational conditions in a real environment certainly make an accurate design process challenging. For example, it would be of little use to design an artificial leaf that performs as desired in a wind tunnel with laminar air flow. Although such experiments could give information on the dynamics of the system, the conditions differ totally from those that would be found outdoors, where branches, neighboring leaves, neighboring plants, and the variable nature of the wind introduce less predictable behavior. Moreover, plants can acclimate to mechanical loads, such as the additional mass of the harvester, by changing the geometric and mechanical properties of the tissue. Such thigmomorphogenetic changes are only observed in plants after a few weeks or months and are complex to predict.

However, keeping this in mind, our approach can provide a basic insight into how the properties of the artificial leaf influence the overall behavior of the harvester. The core outcome of the simulations, as summarized in **Figure 6**, elucidates the role played by the properties of the artificial leaf when combined with a given natural leaf. The mass and stiffness of the artificial leaf affect the dynamic behavior of the plant-hybrid system, and they can be used to tune its motion behavior and to adapt it to the most probable wind speed. Other parameters that could be varied would be the shape of the artificial leaf and, of course, the plant leaf. Yet, we have already observed limitations in the approach presented here. The implemented model suggests that for an average wind speed of up to 4 m/s, regular flapping is unlikely: the artificial leaf seems to move in phase with the *N. oleander* leaf and thus in constant contact. In contrast, the experiments performed at an air flow of ~4 m/s show the occurrence of contact and separation motion and the resulting voltage peaks of the converted energy (**Supplementary Video S2**). This is caused by the flexibility of the laminae, which allows the terminal parts of the leaves to collide while the petioles are indeed in contact (as correctly obtained by the simulation). Separation also occurs between the two laminae; separation of the charges produced on the two laminae occurs, leading to electrostatic induction of the charges on the separated surfaces into the corresponding electrode and causing the measured electrical signals.

As expected, the model predicts that impacts between the two leaves will occur for a lighter, more compliant artificial leaf. This could be achieved by reducing the mass and the stiffness by 40% (case {*µ* = 0.6, *κ* = 0.6}) for which the two leaves collide at a relatively low wind speed (5 m/s), as denoted by the corresponding petal in **Figure 6**, colored in pink. Nevertheless, such leaves may become easily unstable at greater wind speeds, leading to potential damage of the thin electrode layer in the artificial leaf, as explained above (and represented by the two light-blue petals obtained for U = 6 and 8 m/s). Other, less brittle electrode materials could overcome this limitation. On the contrary, it is convenient to maintain the current stiffness while reducing the mass; in this case {*µ* = 0.6, *κ* = 1} turns out to achieve the desired contact-separation behavior under all the wind speeds displayed in **Figure 6**, and shown by the animation in the **Supplementary Video 3**. This allows us to adapt the artificial component to a given plant behavior.

Certainly, the presented model is affected by limitations that could be overcome in the future: above all, it is crucial to increase the number of degrees of freedom to model the deformability of the laminas and the torsional oscillations (as, for example, done for leaves in Shao et al. (2012) and Jiang et al. (2021)). For the latter, it is necessary to introduce the aerodynamic moment in the equations, for which knowledge of the position of the center of pressure on the leaf is required. This could be obtained by a more complex experimental analysis of the dynamics, for example by multi-angle high speed video analysis of the dynamic motions. Another important aspect concerns the dynamics of the branches to which the leaves are attached. As suggested, for example, in Tadrist et al. (2018), there is a critical wind speed for each plant, a threshold above which the dynamics of the plant is no longer dominated by the individual leaves but by the oscillations of the branches. This will then affect and possibly dominate the motion of the individual leaves.

Nevertheless, this model provides the first design tool for artificial devices for operation on plants under wind dynamics. This is especially interesting for designing biohybrid (and bioinspired) mechanical energy harvesters that use the wind motion of the leaf to produce electricity. For example, it can be easily explored how the natural frequency of the artificial leaf can be tuned by dimensions, thickness, multilayer composition, and composite stiffness to achieve, for example, higher impact frequencies for plant leaves of given properties. At the same time, information on the maximum weight of the artificial structure and the force exerted on the leaf under additional wind actuation was determined and used to predict under which conditions leaf damage may occur. Beyond energy harvesting, a model as described here could also give information on how other devices, such as, e.g., leaf sensors for agriculture, behave under the action of wind.

Although our tests have been done with *N. oleander*, we believe that the approach can be easily applied to different species and that a straightforward characterization of the leaf’s oscillations under gravity, together with its dimensions, is sufficient to obtain a good estimation of the overall dynamic behavior. The detailed validation of further species will be part of an extended future investigation.

## Conclusions

These are the first steps towards a model for dimensioning and designing dynamic artificial structures operating on unstructured objects like plant leaves, particularly for the case of mechanical energy harvesters exploiting leaf motion. From the relatively straightforward quasistatic tests of plant tissue, we could derive key parameters to feed into the model. This then allows one to estimate the dynamic behavior of the leaves in the wind as well as the possible petiole damage in combination with an artificial device installed on them. In detail, the tool enables us to better dimension artificial devices and select promising prototypes before experimental validation in a manner that they a) do not harm the tissue by overloading it and b) in the case of the described mechanical energy harvesters, create a motion pattern that could prompt a better performance by optimizing oscillations and thus electricity-generating contact-and-release events. In the future, the model should be tested with and adapted to other plant species, depending on their potential for biohybrid applications. Moreover, it should be extended to account for more complex behaviors and petiole properties, including flexibility of laminae, leaf torsion of both artificial and natural parts, by adding more degrees of freedom. The increasing demand for devices that produce clean energy and monitor plant growth and ecosystems could benefit from the development of such a model, especially when devices will be operated on plant leaves exposed to natural conditions like wind.

## Data availability statement

The code and raw data supporting the conclusions of this article will be made available by the authors, without undue reservation.

## Author contributions

GN developed the mechanical model, analyzed the data, and wrote the manuscript. FM performed experimental tests, analyzed data, and wrote the manuscript. BM wrote the manuscript. All authors contributed to the article and approved the submitted version.

## Funding

This work was funded by the project GrowBot, the European Union’s Horizon 2020 Research and Innovation Programme under Grant Agreement No. 824074.

## Acknowledgments

The authors acknowledge the kind help of Serena Armiento with setting up the high-speed camera recordings.

## Conflict of interest

The authors declare that the research was conducted in the absence of any commercial or financial relationships that could be construed as a potential conflict of interest.

## Publisher’s note

All claims expressed in this article are solely those of the authors and do not necessarily represent those of their affiliated organizations, or those of the publisher, the editors and the reviewers. Any product that may be evaluated in this article, or claim that may be made by its manufacturer, is not guaranteed or endorsed by the publisher.
