# Supplementary material for: Wind dynamics and leaf motion: Approaching the design of high-tech devices for energy harvesting for operation on plant leaves
Source: Front Plant Sci. 2022 Oct 26;13:994429. doi: 10.3389/fpls.2022.994429 (PMC9644130; doi:10.3389/fpls.2022.994429)
Supplement: Supplementary file 1 [file DataSheet_1.docx]

Supplementary Material

Wind dynamics and leaf motion: approaching the design of high-tech devices for energy harvesting for operation on plant leaves

Fabian Meder^1*^, Giovanna Adele Naselli^1*^, Barbara Mazzolai^1*^

# Experimental analysis of leaf and petiole failure and under an external load acting on the blade


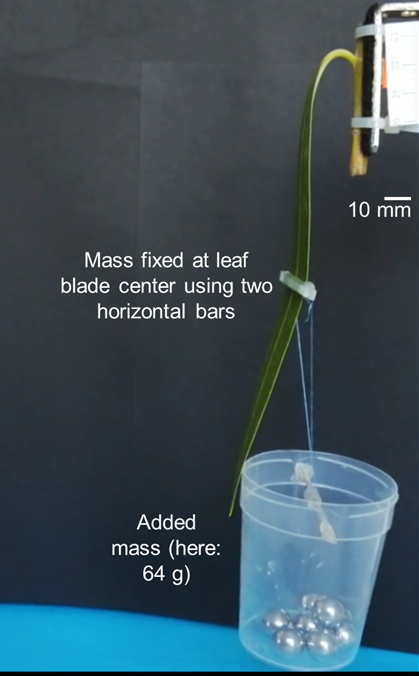


Supplementary Figure 1. Straightforward setup for testing the leaf and petiole resistance to an external load used to determine data shown in Table 1.

# Structural properties of artificial and natural leaf

For this work, we treat the natural petiole as a homogenous, isotropic flexible beam, with semicircular cross section as shown in the Main Text. For the artificial petiole, we show that its layered structure can be exemplified, in the model, by the layer of PET only.

For both leaves, the first modeling stage consists in their discretization as a series of beam elements, whose end points are called nodes. The model is two dimensional since we consider the leaves as systems performing only planar bending.

To summarize, the properties of the petiole appear in the so-called stiffness matrix, $\mathbf{K}$, that relates the nodal displacements$\boldsymbol{u}$and the forces $\boldsymbol{F}$ applied on the nodes. All these quantities are defined in a global system of coordinates, which in this case is assumed as shown in Figure 1b in the Main Text. To account for large deflections of the petioles, such forces are applied incrementally:

|  | $\boldsymbol{\Delta}\boldsymbol{u}_{\boldsymbol{i}}\boldsymbol{=}\mathbf{K}_{\mathbf{i-1}}\left( \boldsymbol{u} \right)^{\boldsymbol{-1}}\boldsymbol{\Delta}\boldsymbol{F}_{\boldsymbol{i}}$ | S 1 |
| --- | --- | --- |

For the static analysis, the forces collected in the array $\boldsymbol{F}$ are the concentrated on the last node (that is, the node at which there is the transition from the petiole to the lamina) and consist of the weight of the lamina and the moment due to the distance between the node and the center of mass of the petiole (taken at the geometrical center). The subscript *i* denotes the step at which the increment of $u$ is calculated, using the stiffness matrix computed based on the results obtained from the previous step. At the final step, the total displacements result from the sum of all the computed increments.

## Natural leaf

To account for the effective shape of the oleander petioles, we model it as a precurved beam, spanning an angle of 45°. As said, we model its deformation by a nonlinear matrix structural analysis (NMSA): all elements have semicircular cross section, with diameter $d=2.8$mm; the free length of the petiole is 8mm; the length of the lamina is 130mm; and the total mass of the leaf is 0.73g.

To ensure that the deflection is simulated up to its maximum, the load applied at the last node (the free end) has been set equal to 8 times the mass of the leaf. The angle $\theta_{E_{N}}$ is computed as the orientation angle of the last segment of the discretization. Supplementary Figure 2 shows the modelled deflection, used to find the relation between $\theta_{E_{N}}$ and $\varphi_{N}$.


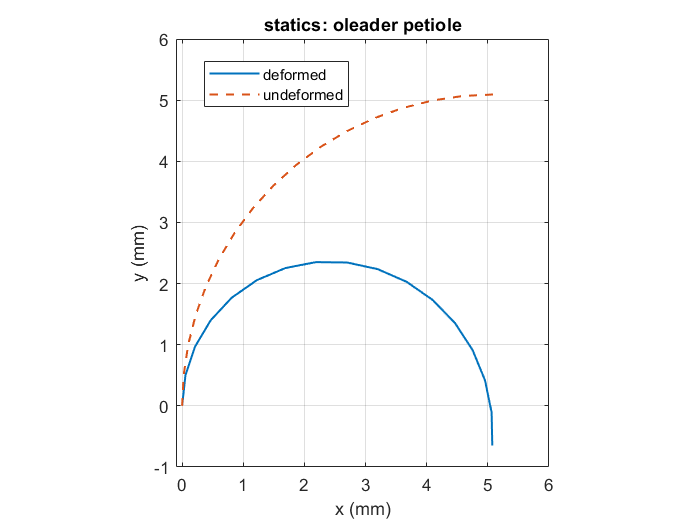


Supplementary Figure 2. Static deflection of the natural petiole as computed by NMSA.

## Artificial leaf

The same procedure using NMSA is adopted for the artificial leaf, which is initially defined as a flat component (its undeformed configuration lies on the x-axis). The result is shown in the figure below.


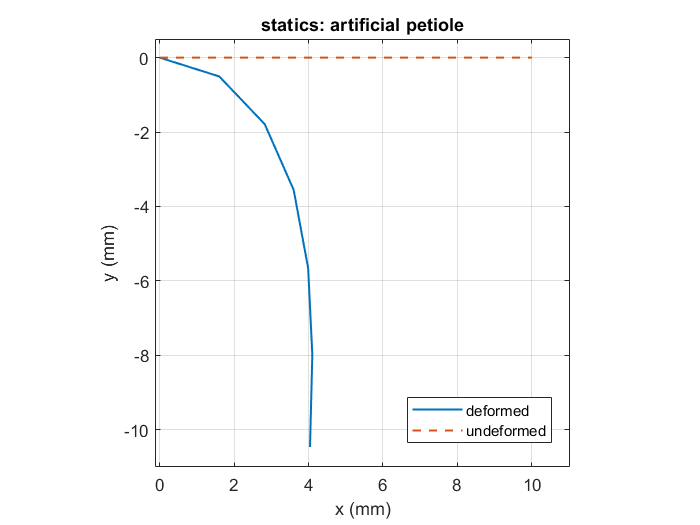


Supplementary Figure 3. Static deflection of the artificial petiole as computed by NMSA

# Modelling of the impact between leaves

As briefly mentioned in the main text, to model the contact between leaves we implement a penalty method, imposing that the following condition is satisfied at any time $t$:

|  | ${f=\gamma}_{N}\left( \varphi_{N} \right)-\gamma_{A}\left( \varphi_{A} \right)\leq0$ | S 2 |
| --- | --- | --- |

with $\gamma_{N}$ and $\gamma_{A}$ as defined in the Main Text. The system of equations of motions is hence written as:

|  | $\left\{ \begin{matrix} {J_{t_{N}}\ddot{\varphi}_{N}+R_{N}\dot{\varphi}_{N}+K_{t_{N}}\varphi_{N}+\eta\frac{\partial f}{\partial\varphi_{N}}=M}_{T_{N}}(\phi_{N}) \\ {J_{t_{A}}\ddot{\varphi}_{A}+R_{A}\dot{\varphi}_{A}+K_{t_{A}}\varphi_{A}+\eta\frac{\partial f}{\partial\varphi_{A}}=M}_{T_{A}}(\phi_{A}) \end{matrix} \right.$ | S 3 |
| --- | --- | --- |

where the terms $M_{T}$ denote the sum of the two moments to which the petiole is subject, due to the gravity and to action of the wind, and $\eta$ represents the so-called penalty factor, defined as

|  | $\eta=\left\{ \begin{matrix} k\cdot f\left( q \right) if f\left( q \right)>0 \\ 0 if f\left( q \right)\leq0 \end{matrix} \right.$ | S 4 |
| --- | --- | --- |

We have found that setting $k=100$ Nm provides satisfactory results for the specific problem addressed here.

The angle of attack $\psi$ for the generic leaf is computed as follows:

|  | $\psi\left( t \right)=\tan^{-1} \frac{v_{C}\left( t \right)}{U\left( t \right)}+\psi_{0}$ | S 5 |
| --- | --- | --- |

Where $v_{C}$ denotes the linear velocity of the centre of the lamina, and therefore it depends on the angular velocity $\dot{\varphi}$, and $\psi_{0}$ denotes the initial angle of attack.

# Stress-strain analysis of the artificial leaf petiole

A stress-strain analysis of the petiole is interesting to evaluate the stability of the thin electrode layer in the artificial leaf as it allows predicting the maximal bending curvature before this electrode cracks leading to a loss of conductivity and reducing the capability to harvest charges on the artificial leaf. Indeed, the ~100 nm thick ITO electrode is the most sensitive compartment of the multilayered artificial leaf having a Young’s modulus of ~100 GPa at room temperature and an ultimate strain of 0.022 (Cairns et al., 2001). To perform the stress strain analysis, the multimaterial, layered artificial leaf must be mathematically transformed into an equivalent cross-section made of only one material (here modelled for the PET layer alone as it is the determining element of the multilayer). Bending of the leaf results in an arc shape, covering an angle $\alpha$ with the radius of curvature $\rho=l_{P_{A}}/\alpha$. Assuming that the strain varies linearly along the cross-section, the maximum tensile strain can be computed as $\varepsilon_{MAX}={t_{P_{A}}}/{2\rho}$. As there is no load longitudinally applied on the leaf, we can state that an $\varepsilon_{MAX}<0.022$ would ensure mechanical integrity of a 100 nm thick ITO electrode, and, therefore, its conductivity. The analysis shows that if the artificial leaf’s petiole bends along a circular arc with an angle equal to 180°, the ITO layer reaches a strain of $\varepsilon_{MAX}=$ ~0.031. This value is above the ultimate strain and suggests damage of the ITO electrode. Hence, if the artificial leaf flips backwards, for example in strong winds, it could damage the electrode. A design solution for this could be reducing the thickness of the PET layer, hence reducing $\varepsilon_{MAX}$. Also using cross-sectional geometries with a higher axial second moment of area than the rectangle of the artificial petiole could avoid back-flipping. Furthermore, it could turn out necessary to build the composite leaf in such a way that the ITO layer is placed on the neutral plane or replaced by another electrode type, with greater elongation at break. Yet, as we will see in the following dynamic analysis, strong bending by flipping backwards of the artificial leaf may only occur for certain designs and operational conditions

# Dynamics of the coupled leaves under the action of the airflow at 4 m/s

Supplementary Figure 4 shows the vertical displacements of the markers visible in the Supplementary Video S2, generated by the airflow at average speed 4 m/s.


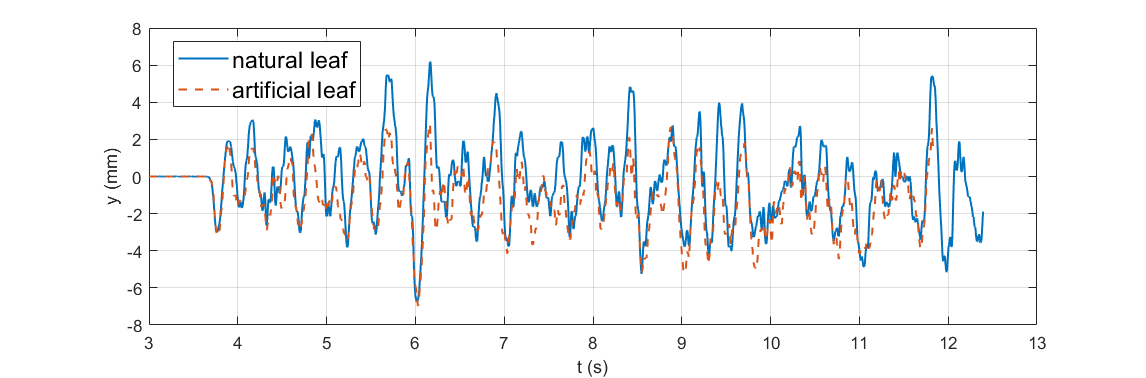


Supplementary Figure 4. Vertical displacement (y) vs. time (t) of the center of lamina of the natural and the artificial leaf, under the airflow at 4 m/s. The curves are obtained by tracking the trajectories from the Supplementary Video S2
